# An EPR Study on the Interaction between the Cu(I) Metal Binding Domains of ATP7B and the Atox1 Metallochaperone

**DOI:** 10.3390/ijms21155536

**Published:** 2020-08-02

**Authors:** Michael Zaccak, Zena Qasem, Lada Gevorkyan-Airapetov, Sharon Ruthstein

**Affiliations:** Department of Chemistry, Faculty of Exact Sciences, Bar Ilan University, Ramat-Gan 5290002, Israel; m_z_91b@hotmail.com (M.Z.); zena_khb94@hotmail.com (Z.Q.); ladaga77@gmail.com (L.G.-A.)

**Keywords:** copper metabolism, metal binding domains, Atox1, ATP7B, EPR, DEER

## Abstract

Copper’s essentiality and toxicity mean it requires a sophisticated regulation system for its acquisition, cellular distribution and excretion, which until now has remained elusive. Herein, we applied continuous wave (CW) and pulsed electron paramagnetic resonance (EPR) spectroscopy in solution to resolve the copper trafficking mechanism in humans, by considering the route travelled by Cu(I) from the metallochaperone Atox1 to the metal binding domains of ATP7B. Our study revealed that Cu(I) is most likely mediated by the binding of the Atox1 monomer to metal binding domain 1 (MBD1) and MBD4 of ATP7B in the final part of its extraction pathway, while the other MBDs mediate this interaction and participate in copper transfer between the various MBDs to the ATP7B membrane domain. This research also proposes that MBD1-3 and MBD4-6 act as two independent units.

## 1. Introduction

Copper is an essential trace metal ion, involved in numerous enzymatic and electron transfer reactions of the cell. However, its ability to cycle between two oxidation states can result in the formation of reactive oxygen species that potentially can cause harm and cell death [1,2]. Therefore, copper homeostasis is highly controlled in all living species. In the human cell, the main copper import transporter Ctr1, and the two P-type ATPase exporters ATP7A and ATP7B, regulate copper concentration [3,4,5]. The metallochaperone Atox1 is responsible for shuttling copper ions from Ctr1 to ATP7A/B. ATP7A/B play two roles: (1) transferring copper to the secretory pathway, where Cu(I) can be incorporated into copper-dependent enzymes, and (2) removing excess Cu(I) from the cell. Mutations in the ATP7A and ATP7B protein genes lead to the disruption of normal Cu(I) distribution, causing serious neurological disorders in humans; these are known as Menkes disease and Wilson’s disease, respectively [6,7,8,9].

**Scheme 1:**
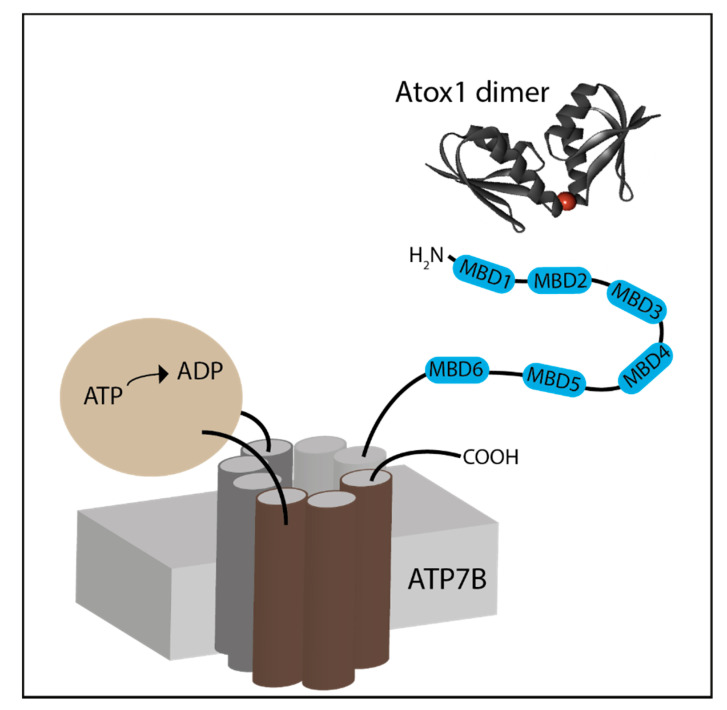
An illustration of Atox1 dimer (PDB 3IWX) and ATP7B transporter.

ATP7A/B are composed of eight transmembrane segments and two large cytosolic domains: the catalytic ATP hydrolyzing domain and the N-terminal copper-binding domain [3]. The N-terminal domain is responsible for shuttling copper ions from Atox1 to the transmembrane domain of ATP7A/B (Scheme 1) [10]. The N-terminus is composed of six metal binding sub-domains (MBDs) containing the MXCXXC motif, which is known to coordinate Cu(I) via two cysteine residues. Each ∼70-residue MBD has a ferredoxin-like fold with a compact βαββαβ structure. The complete N-terminus is about 630 residues long, of which roughly 200 residues belong to the inter-domain linkers. The longest linker is located between MBD4 and MBD5; it consists of 46 residues. The lengths of the other linkers are shorter: 29 residues between MBD3 and MBD4, 24 residues between MBD2 and MBD3, and 13 residues between MBD1 and MBD2. The shortest linker is between MBD5 and MBD6, which consists of only six residues. Indeed, NMR characterization of the MBDs suggests that MBD1-4 are much more dynamic than MBD5-6 [11,12,13]. Moreover, NMR studies using nanobodies on MBD1-6 revealed a transient inter-domain interaction between MBD2 and MBD3 [14]. Molecular dynamics (MD) simulation suggests that MBD1-3 and MBD5-6 act as two independent units, whereas MBD4 has little interaction with each of them [15]. Yeast cell experiments showed that MBD1-3 acts as a regulatory unit, whereas MBD5-6 is essential for ensuring proper copper transfer [16]. The importance of MBD5-6 in copper transfer is also linked to Wilson’s disease, wherein most of the mutations in the N-terminus domain of ATP7B were reported in this unit [17,18].

Atox1 is a dimer in solution; each monomer holds a structure similar to the MBD of ATP7A/B. Direct protein–protein interactions between the Atox1 monomer and MBDs are essential for the delivery of Cu(I) to the MBDs [19]. In principle, Atox1 can deliver Cu(I) to all MBDs in vitro [20]. However, not all MBDs are equivalent in terms of receiving Cu(I) from Atox1. Magnetic resonance spectroscopy and computation studies demonstrated that Atox1 can form a stable adduct with MBD1, MBD2 and MBD4, which are more solvent-exposed; however, not with MBD3 and MBD5-6 [12,21,22,23]. Although MBD5-6 are critical domains for proper copper transport to the membrane domain [24], it is still unclear how copper is transferred to these domains.

**Figure 1:**
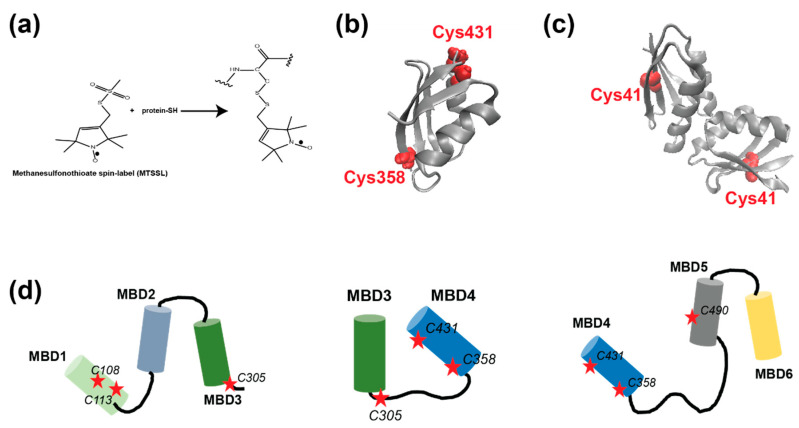
(**a**) The scheme of the site-directed spin-labeling of the methanesulfonothioate (MTSL) nitroxide radical to cysteine residues. (**b**) The MBD4 structure, PDB 2ROP; the cysteine residues accessible for spin-labeling are denoted in red. (**c**) The Atox1 dimer structure, PDB 3IWX; the cysteine residues accessible for spin-labeling are denoted in red. (**d**) Cartoons of the MBDs of ATP7B studied here. The red stars denote the cysteine residues, which were spin labeled.

Recently, we conducted electron paramagnetic resonance (EPR) spectroscopy experiments in order to better understand the interaction between the Atox1 metallochaperone and MBD3-4 as a function of Cu(I) binding [23,25]. The power of EPR spectroscopy lies in its ability to target conformational changes in solution, without limiting the protein size or the system. However, paramagnetic centers should be presented when a diamagnetic system is studied. Proteins are best targeted using site-directed spin labeling (SDSL), with a nitroxide spin-label (Methanesulfonothioate, MTSL) [26,27] that is linked to cysteine residues (Figure 1a). Using SDSL and EPR spectroscopy, we showed that the dimerization of Atox1 breaks upon interaction with MBD3-4. Atox1 can also interact with MBD3-4 in the absence of copper. We also showed that the Atox1 monomer interacts with MBD4, and not with MBD3, in a face-to-face manner, where two helices (α2), one from MBD4 and the second from Atox1, point toward each other. The organization of the six MBDs during copper transfer is still unknown, due to lack of structural information. Here, we applied EPR spectroscopy to MBD1-3 and MBD4-6, exploring their interaction with Atox1. Further, we compared these results with our previous ones from MBD3-4 [23,28].

## 2. Results

### 2.1. Probing the Interaction between the MBDs of ATP7B and the Metallochaperone Atox1 by CW-EPR at RT

**Figure 2:**
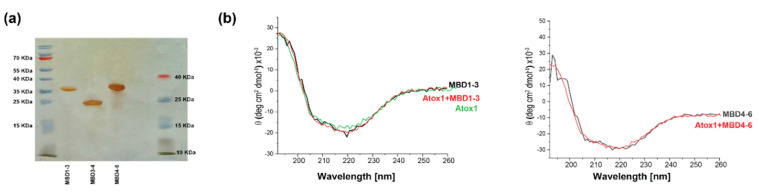
(**a**) SDS-gel picture of MBD1-3 (34.9 KDa), MBD3-4 (21.6 KDa) and MBD4-6 (29.2 KDa). (**b**) CD spectra of MBD1-3 and MBD4-6 in the absence and presence of Atox1, and of Atox1 alone.

**Figure 3:**
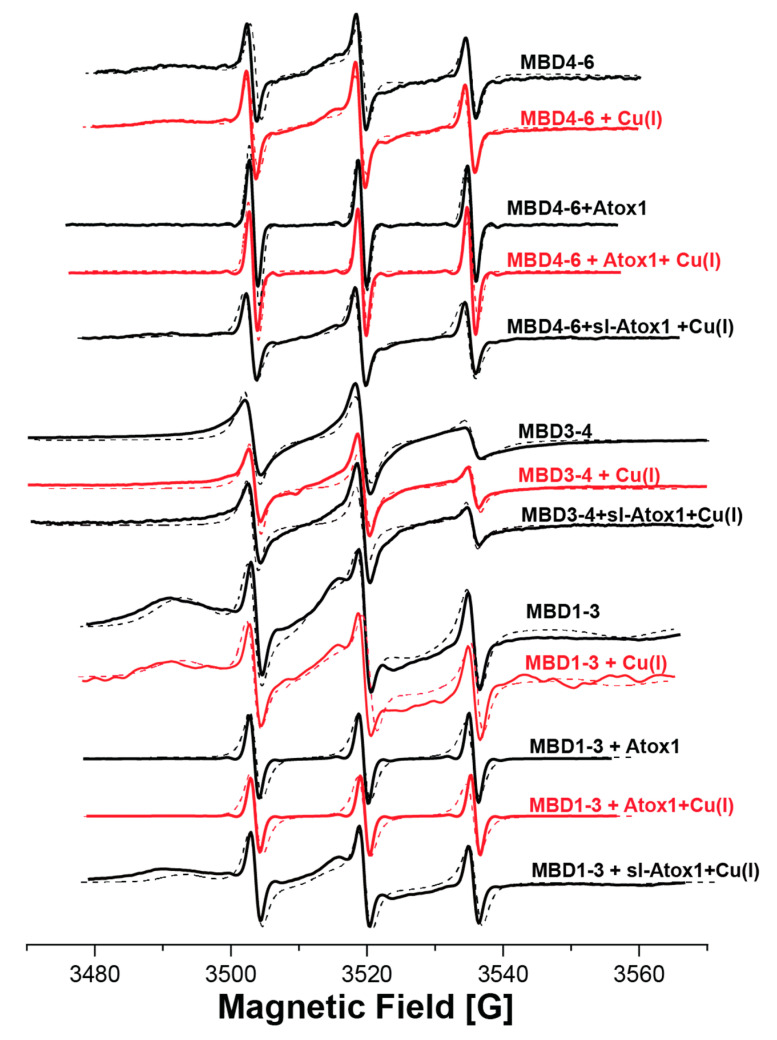
Room-temperature continuous wave (CW) EPR spectra (solid lines) of the various MBDs in the presence and absence of Cu(I), Atox1, and sl-Atox1 (spin-labeled Atox1). The dotted lines denote the simulated spectra using the parameters listed in Table 1. The CW-EPR spectra of MBD3-4 were already reported in reference [23].

EPR spectroscopy has emerged as an excellent tool for probing protein–protein interaction, since it does not require crystallization and does not depend on protein size. EPR can be measured in buffer solution, and even weak interactions between proteins can be detected [29]. EPR’s strength lies in its sensitivity to both atomic level changes and nanoscale fluctuations. Continuous wave (CW) EPR can determine the dynamics of protein chains. The combination of CW-EPR with site-directed spin labeling has become widely used in biophysical research [26,30,31,32,33]. Here, we explored the interaction between various MBDs of ATP7B and the Atox1 metallochaperone. The structure of MBD4 and the cysteine residues that are available for spin labeling are shown in Figure 1b. The structure of the Atox1 dimer, and the cysteine that is available for spin labeling at each of its monomers, are presented in Figure 1c. Figure 1d presents the MBDs studied in this research, and the cysteine positions that were spin-labeled with MTSL. MBD1-3 were labeled at C108 and C113 located on helix α2 of MBD1, and at C305 of MBD3 (located in the loop between β3 and α2 of MBD3). MBD3-4 were spin labeled at C305 (located in the loop between β3 and α2 of MBD3), C358 and C431 (located in β1 and β4 of MBD4, respectively). MBD4-6 were spin-labeled at C358 and C431 on MBD4, and at C490 (located in β1 of MBD5). After spin labeling, the spin label attached to the cysteine (Cys) residues is termed R1. Figure 2a shows the SDS-gel picture of the various proteins studied here: MBD1-3, MBD3-4 and MBD4-6. Figure 2b shows the CD spectra of MBD1-3 and MBD4-6 in the presence and absence of Atox1, showing no change in the secondary structure of these domains when interacting with the Atox1 metallochaperone. Figure 3 shows the CW-EPR spectra of the various MBDs in the presence and absence of Cu(I) and the Atox1 metallochaperone. Atox1 was added to the protein solution as a wild-type non spin-labeled protein (here it was termed “Atox1”), and also spin-labeled at the C41 position (termed “sl-Atox1”) in a ratio of 1:1 (Atox1:MBD). The CW-EPR spectra were simulated using the slow-motion theory designed by Freed and co-workers [34], as implemented in the easyspin toolbox [35], and are denoted as dotted lines in Figure 3; the parameters of the simulations are presented in Table 1. For all spectra, the g-tensor used was constant g = [2.0087 2.006 2.0022]. The CW-EPR spectra of Atox1, in the absence and presence of Cu(I), were already reported in our previous study [36]. Atox1 was simulated with a single species with a correlation time of 2·10^−9^ s, and an isotropic electron–electron interaction (ω_ee_) value of 4 MHz. The addition of Cu(I), in a ratio of Atox1:Cu(I) of 1:3, did not affect the CW-EPR spectrum. The CW-EPR spectrum of MBD3-4 is very similar to the CW-EPR spectrum of Atox1 dimer; it was also simulated with a dominant species (98%) and with a correlation time of 2·10^−9^ s, and an isotropic electron–electron interaction (ω_ee_) value of 5 MHz, where 2% of the spin-labels were simulated without an isotropic electron–electron interaction, but with the same dynamics. The addition of Cu(I) to MBD3-4 solution results in a spectrum with slightly higher dynamics, and a reduction in the isotropic electron–electron interaction, as reported before [23]. The addition of sl-Atox1 to MBD3-4 in the presence of Cu(I) did not affect the CW-EPR spectrum. The CW-EPR spectra of both MBD1-3 and MBD4-6 are different from those of MBD3-4 and Atox1. The CW-EPR spectra of MBD1-3 and MBD4-6 are characterized by two species, where the correlation time of the dominant species (90% and 84% respectively) is 1·10^−6^ s (approaching the rigid limit, which corresponds to low dynamics), and the second species was simulated with a correlation time of 1·10^−10^ s. Both species were simulated in the presence of an isotropic electron–electron interaction (ω_ee_) value of 5–6 MHz. The addition of Cu(I) to MBD1-3 and MBD4-6 hardly affected the CW-EPR spectra. However, the addition of non-spin-labeled Atox1 to the solution resulted in an EPR spectrum characterized by high dynamics of the spin labels. This suggests that the spin labels at MBD1 and MBD4 were largely affected by the interaction with Atox1. The addition of Cu(I) to the MBDs + Atox1 solution hardly affected the CW-EPR spectrum for any of the MBDs. Interestingly, when adding spin-labeled Atox1 (sl-Atox1) to the solution, the less dynamic species increased. Since the dynamics of a spin label bound to the C41 (C41R1) of Atox1 is relatively high, the only explanation for this is that when spin-labeled Atox1 interacts with MBD1-3 and MBD4-6, the dynamics of C41R1 is reduced, meaning that Atox1 is trapped between the various MBDs. This is in agreement with our previous findings [23], where we suggested that the Atox1 monomer interacts with MBD3-4 only in a face-to-face manner with MBD4, and that it is not affected by the presence of MBD3. Here, the dynamics of the spin-labeled C41R1 position of Atox1 are not affected and not reduced, since there is no other MBD nearby. However, for MBD4-6, the Atox1 monomer points to MBD4 in a face-to-face manner, and to MBD5 in a back-to-face manner. Here, the spin label at the C41R1 position is affected by the presence of MBD5. Since similar behavior was obtained also for MBD1-3 when interacting with Atox1, the CW-EPR data suggest that the Atox1 monomer interacts in a face-to-face manner with MBD1, and in a back-to-face manner with MBD2.

### 2.2. Probing the Interaction between the MBDs of ATP7B and the Metallochaperone Atox1 by Distance Pulsed EPR Measurements (DEER)

**Figure 4:**
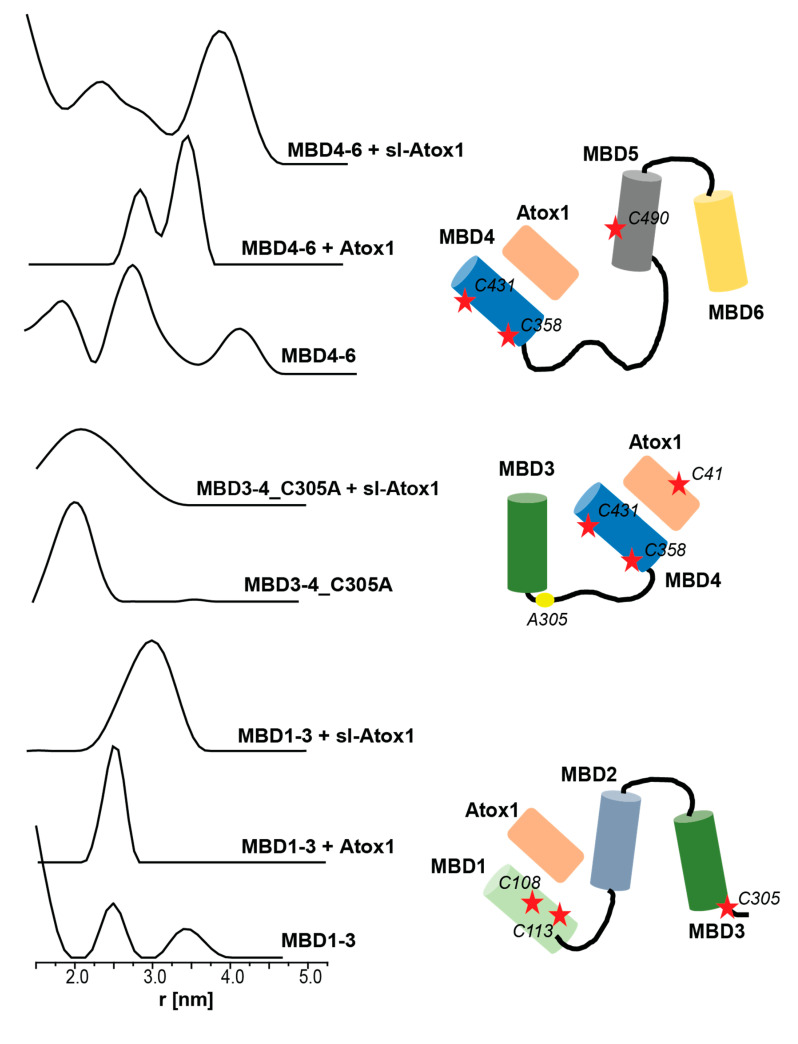
**Double electron electron resonance** (DEER) distance distribution functions for spin-labeled MBD1-3, MBD3-4_C305A and MBD4-6, in the absence and presence of Atox1 and sl-Atox1. The DEER distance distributions of MBD3-4_C305A were already reported in reference [23]. The figure also presents the spin-label positions in the various MBDs and Atox1.

To further explore the conformational changes that the various MBDs and Atox1 experience upon interaction, double electron–electron resonance (DEER) experiments were carried out. DEER is a pulsed EPR experiment that can target the distance distribution between two or more paramagnetic centers (here spin labels), where the distances range from 1.5 nm to 8.0 nm [37]. Figure 4 shows the DEER distance distribution functions obtained for the various MBDs in the absence and presence of Atox1. DEER experiments performed on MBD3-4 and MBD3-4_C305A in the presence of sl-Atox1 were already reported by us earlier, and showed a similar broad distance distribution function of between 1.5 and 3.5 nm [23], suggesting that Atox1 interacts only with MBD4, and that no conformational changes in MBD3 occurred while interacting with MBD4. MBD4-6, labeled at C358R1, C431R1 from MBD4, and C490R1 from MBD5 showed three distance distribution functions. The distribution around 2.0 ± 0.4 nm corresponds to the distance between C358R1 and C431R1 in MBD4, whereas the two other distributions, 2.7 ± 0.4 nm and 4.0 ± 0.3 nm, correspond to the C431R1–C490R1 and C358R1–C490R1 distances. Interestingly, when adding non spin-labeled Atox1, a bimodal distance distribution function between 2.5 and 3.5 nm is observed. However, this can occur only if the distances between all spin labels (C358R1, C431R1 and C490R1) have changed, indicating conformational changes that occur both within MBD4 as well as between MBD4 and MBD5. Based on the difference in the intensity of each distribution, we suggest that the distribution of 3.4 ± 0.2 nm corresponds to the C358R1–C490R1 and C430R1–C490R1 distances, and that the distribution of 2.8 ± 0.2 nm corresponds to the C358R1–C431R1 distance. C358 and C431 are located in the β1 and β4 of MBD4; the change in the distribution between C358R1 and C431R1 upon interaction with Atox1 was not detected when the DEER experiment was performed on MBD3-4. This suggests that the presence of MBD5-6 caused structural changes in MBD4, in order to get it closer to MBD5-6, manifested by a decrease in the distance between MBD4 and MBD5, which will eventually allow copper to be transferred between these three units. This is consistent with the CW-EPR spectrum that also detected large changes in the dynamics of the spin labels after an interaction between MBD4-6 and Atox1. When spin-labeled Atox1 (sl-Atox1) is added to MBD4-6, the distance distribution around 2.8 nm gets broader and additional distribution around 3.8 nm appears, owing to the presence of the additional spin-label at the C41R1 position of Atox1. Since more than one distance distribution appears in the presence of sl-Atox1, this confirms as well that Atox1 closely interacts with MBD4.

MBD1-3 was spin-labeled at C108 and C113 located on helix α2 of MBD1, and on C305 of MBD3 (located in the loop between β3 and α2 of MBD3). The distance distribution suggests a small distance distribution below 1.8 nm, which probably corresponds to the C108R1–C113R1 distance, and to two distributions, 2.5 ± 0.4 nm and 3.4 ± 0.4 nm, which we assigned to the C113R1–C305R1 and C108R1–C305R1 distances. Intriguingly, after adding Atox1 to MBD1-3, the DEER distance distribution is characterized by a single, comparably narrow, distance distribution of 2.5 ± 0.25 nm. This proposes that once Atox1 interacts with MBD1-3, either a compact structure is formed with a unique average distance between all three spin labels, or that MBD3 dissociates from MBD1 with a distance that is much larger than 5.0 nm, which could not be detected by the DEER measurement (τ2 was set to 1400 ns). The increase in the dynamics of the CW-EPR spectrum suggests that the latter is correct, and that MBD3 is distanced from MBD1, which results in an increase in the dynamics of the C305R1 spin label. C108R1 and C113R1 are located on α2 of MBD1, which is proposed to interact with α2 of Atox1 in a face to face manner. The increase in the distance between these two spin labels confirms this interaction, where the orientation of the spin labels changes upon Atox1-MBD1 interaction. The DEER distance distribution obtained after sl-Atox1 was added to MBD1-3 is characterized by a broader distance distribution of between 2.2 and 3.8 nm, confirming that Atox1 interacts with MBD1 and that when Atox1 is spin-labeled at C41R1, an interaction occurs between this spin label and the C108R1 and C113R1 spin labels.

## 3. Discussion

In the last decade, distinct biophysical tools have been used to characterize the interaction between Atox1 and the six MBDs of ATP7A/B, sometimes leading to contradictory conclusions [38,39,40]. The interaction between Atox1 and MBDs is essential for proper Cu(I) transfer to the ATP7B membrane domain. In theory, Atox1 can interact as a monomer with each of the MBDs. However, experimental and computational research suggests that Atox1 can form a stable adduct with MBD1, MBD2 and MBD4, which are more solvent exposed; however, not with MBD3 and MBD5-6 [12,21,22,23]. NMR experiments, yeast cell experiments and MD simulations suggest that MBD1-4 are much more dynamic than MBD5-6 [11,12,13,15,16]. These studies also proposed that MBD4 has little interaction with MBD1-3 and MBD5-6. The importance of MBD5-6 in copper transfer is also linked to Wilson’s disease, wherein most of the mutations in the N-terminus domain of ATP7B were reported in this unit [17,18].

Here, we applied EPR spectroscopy to better understand the interaction between the MBDs of ATP7B and the Atox1 metallochaperone. EPR can target the conformational changes that proteins undergo in solution while interacting with a partner protein. The following units have been expressed, purified and spin-labeled: MBD1-3, MBD3-4 and MBD4-6. The RT CW-EPR experiments suggest that the dynamics of spin labels at MBD1 and MBD4 are reduced by the presence of their partner units MBD2-3 and MBD5-6, respectively, whereas when Atox1 interacts with just MBD3-4, no change in the dynamics of the spin labels is observed. When Atox1 is introduced into a solution of MBD1-3 and MBD4-6, a change in the dynamics of all spin labels is detected, suggesting a close interaction between the MBDs and Atox1. It has been shown that when sl-Atox1 is introduced to the MBDs solution, Atox1 closely interacts with MBD1 and MBD4, and an increase in the dipolar interaction and a reduction in the dynamics of C41R1 located in Atox1 occurs. This confirms that Atox1 is trapped between MBD1-2 and MBD4-5. These results were supported by pulsed EPR measurements. The experiments confirmed that Atox1 has a preference to interact with MBD1 and MBD4 in a face-to-face manner. When Atox1 interacts with MBD1, a change in the orientation of MBD1-3 occurs, suggesting that MBD1-3 change their conformation, and that MBD3 distances itself from MBD1-2. When Atox1 interacts with MBD4-6, a change in the conformation of MBD4 and MBD5 is also observed; however, when Atox1 interacts with MBD3-4, no change in the orientation of MBD3 is reported [23]. The latter confirmed a close interaction with MBD4, where Atox1 points to MBD4 in a face-to-face manner and to MBD5 in a face-to-tail manner. The flexibility of MBD1-6 was also reported by NMR measurements; MBD1-6 is thought to play an essential role in the copper transfer mechanism [41]. NMR experiments also suggest that MBD4-6 have an intended mobility in solution, and that MBD2-3 seem to work together and undergo changes in their dynamics [14]. A mechanistic model for copper delivery from Atox1 to MBD1-6 was proposed, based on mutagenesis yeast cell experiments, where each time a single cysteine was mutated to serine. This study shows that Atox1 interacts with MBD1-3, where it is thought that Cu(I) is initially loaded to MBD2, then Cu(I) is transferred from MBD3 to MBD6. Another scenario was suggested whereby Atox1 interacts with MBD4, and then Cu(I) is transferred to MBD6 [16]. The EPR performed here supports the conclusion that MBD1-3 and MBD4-6 act as independent units. Our results, however, suggest that Atox1 is trapped between MBD1 and MBD2, and that therefore, Cu(I) is probably first transferred to MBD1 and then to MBD2. We also observed large changes in the conformation of MBD3 in the presence of Atox1. These conformational changes were only detected for MBD1-3 and not for MBD3-4. This indicates that MBD3 transfers Cu(I) either to MBD5-6 or directly to the ATP7B membrane protein, but not to MBD4, whereas MBD4 transfers Cu(I) to MBD5-6.

## 4. Materials and Methods

### 4.1. Cloning, Expression and Purification of ATP7B 1-3; 3-4; 4-6 Metal Binding Domains

The ATP7B 1-3, 3-4, 4-6 metal binding domains’ (MBD1-3, MBD3-4 and MBD4-6) genes were first amplified by PCR using primers containing specific ATP7B MBD sequences and flanking regions that corresponded to the expression vector sequences pTYB12.

Forward primer of ATP7B 1-3 domains:

5′-GTTGTACAGAATGCTGGTCATCAGGTGGCCACCAGCACAGTC -3′.

Reverse primer of ATP7B 1-3 domains:

5′-GTCACCCGGGCTCGAGGAATTTCAAAGAGAAACTTTAAAATTCCCAGGTGG-3′.

Forward primer of ATP7B MBD3-4:

5′-GTTGTACAGAATGCTGGTCATATGAGACCTTTATCTTCTGCTAAC-3′.

Reverse primer of ATP7B MBD3-4:

5′-GTCACCCGGGCTCGAGGAATTTCAGTGGTTTCCAAGAGGGTTAGT-3′.

Forward primer of ATP7B 4–6 domains:

5′-GTTGTACAGAATGCTGGTCATGGCACATGCAGTACCACTCTG-3′.

Reverse primer of ATP7B 4–6 domains:

5′-GTCACCCGGGCTCGAGGAATTTCACTGGGCCAGGGAAGCATGAAAG-3′.

This amplicon was cloned into the pTYB12 vector by restriction-free cloning [23]. pTYB12 is a cloning and expression vector that allows the overexpression of ATP7B as a fusion to a self-cleavable intein tag. The self-cleavage activity of the intein allows the release of ATP7B from the chitin-bound intein tag. The clone was expressed in the *E. coli* strain Origami 2. A stock solution of glycerol was added to the starter and grown at 37 °C reaching an optical density of 0.6–0.8 (OD_600nm_) using terrific broth (TB) medium supplemented with ampicillin and tetracycline as selection factors. The starter was then induced with 1 mM isopropyl-β-d-thiogalactopyranoside (IPTG) at 18 °C overnight. Bacteria were then harvested by centrifugation at 10,000 rpm for 30 min. Then, the pellet was re-suspended in lysis buffer (25 mM Na_2_HPO_4_, 150 mM NaCl, 20 mM PMSF, 1% triton, pH 8.8) and sonicated (10 min of pulse for 30 s of 40% amplitude). Finally, lysate was centrifuged at 14,500 rpm for 30 min and the supernatant was kept.

For purification, the lysate was loaded on the chitin bead column, allowing the ATP7B-intein to bind to the resin through its chitin-binding tag. Resin was washed with 50-column volumes of lysis buffer. Then, 5 mL dithiothreitol (50 mM DTT) was added and the resin was incubated for 48 h at 4 °C to perform self-cleavage of the intein. Elution fractions were collected from the column using the chitin column buffer (pH = 8.8) and checked by 14% Tricine SDS-PAGE.

Atox1 expression and purification are similar to the expression and purification of ATP7B described in a series of publications [36,42,43].

### 4.2. Spin Labeling

The spin-labeling process was performed in the presence of Cu(I) ions in order to prevent spin-labeling of the cysteine residues involved in Cu(I) binding. Before labeling, 10 mM DTT was added to the protein solution and mixed overnight (o.n.) at 4 °C. DTT was dialyzed out using 1 kDa dialysis cassettes (Pierce). Next, 0.25 mg of *S*-(2,2,5,5-tetramethyl-2,5-dihydro-1H-pyrrol-3-yl)methyl methanesulfonothioate (MTSL, TRC) was dissolved in 15 μL Dimethyl sulfoxide (DMSO, Bio lab) and added to 0.75 mL of 0.05 mM protein solution (20-fold molar excess of MTSL). The protein solution was then vortexed overnight at 4 °C. The free spin label and Cu(I) ions were removed by several dialysis cycles over 4 days. The mass of the spin-labeled protein was confirmed by a mass spectrometer, and the concentration was determined by BCA assay.

### 4.3. Cu(I) Addition

For EPR measurements, Cu(I) (Tetrakis (acetonitrile) copper(I) hexafluorophosphate) was added to the protein solution under nitrogen gas to preserve the anaerobic conditions. No Cu(II) EPR signal was observed at any time.

### 4.4. X-band CW EPR Experiments

CW-EPR spectra were recorded using an E500 Elexsys Bruker spectrometer operating at 9.0−9.5 GHz and equipped with a super-high-sensitivity CW resonator. The spectra were recorded at room temperature (292 ± 5 K), at a microwave power of 20.0 mW, a modulation amplitude of 1.0 G, a time constant of 60 ms, and a receiver gain of 60.0 dB. The samples were measured in 1.0-mm quartz tubes (Wilmad-LabGlass, Vineland, NJ, USA). The final spin-labeled protein concentration was between 0.01 and 0.03 mM.

### 4.5. Q-band DEER Experiments

The DEER experiment π/2(ν_obs_) − τ1 − π(ν_obs_) − t′ − π(ν_pump_) − (τ1 + τ2 − t′) − π(ν_obs_) − τ2 − echo was carried out at 50 ± 1.0 K on a Q-band Elexsys E580 spectrometer (equipped with a 2-mm probe head) equipped with AmpQ 50W. A two-step phase cycle was employed on the first pulse. The echo was measured as a function of t′, and τ2 was kept constant to eliminate any relaxation effects. The durations of the observer and the pump π pulse were 24 ns each. The dwell time was 20 ns. τ1 was set to 200 ns and τ2 was set to 900–2200 ns. The observer frequency was about 33.75 GHz, and the pump frequency was set to 60 MHz higher. The samples were measured in 1.6-mm capillary quartz tubes (Wilmad-LabGlass). The data were analyzed using the DeerAnalysis program, with Tikhonov regularization. We optimized the regularization parameter in the L curve by examining the fit of the time domain signal.

### 4.6. Circular Dichroism Characterization

Circular Dichroism (CD) measurements were performed using a Chirascan spectrometer (Applied Photophysics, Leatherhead, UK) at room temperature. Measurements were carried out in a 1-cm optical path length cell. The data were recorded from 190 to 260 nm with a step size and a bandwidth of 1 nm. Spectra were obtained after background subtraction. Circular Dichroism measurements were conducted after dialysis steps were carried out with water.

## 5. Conclusions

In this study, using EPR spectroscopy, we showed that Atox1 preferentially interacts with MBD1 and MBD4. After interaction, the nearby MBDs become closer to Atox1, to allow a transfer of Cu(I) between them. This study also suggests that MBD1-3 and MBD4-6 act as independent units, where both MBD3 and MBD5-6 undergo conformational changes in the presence of Atox1. We suggest that MBD3 transfers Cu(I) to MBD5-6 or directly to the ATP7B membrane protein, but not to MBD4, whereas MBD4 transfers Cu(I) to MBD5-6. This study complements previous studies and sheds additional light on the transfer mechanism of Cu(I) from Atox1 to MBDs of ATP7B.

## Figures and Tables

**Table 1:** Simulated parameters for CW-EPR spectra obtained with the easyspin, chili method, in the presence of two species at a g tensor of [2.0087 2.006 2.0022].

Sample	A-Tensor [MHz] (±1.0 MHz)	τ (Correlation Time (Sec)) (±0.05 × 10^−9^)	Exchange/Dipolar Interaction [MHz] (±0.1 MHz)	% Species (±1.0%)
**MBD1-3**	[25 25 88]	1 × 10^−10^	5.0	10%
	[25 25 92]	1 × 10^−6^	6.0	90%
**MBD1-3 + Cu(I)**	[25 25 88]	1 × 10^−10^	5.0	15%
	[25 25 92]	1 × 10^−6^	6.0	85%
**MBD1-3 + Atox1**	[25 25 88]	1 × 10^−10^	5.0	95%
	[25 25 92]	1 × 10^−6^	6.0	5%
**MBD1-3 + Atox1 + Cu(I)**	[25 25 88]	1 × 10^−10^	5.0	95%
	[25 25 92]	1 × 10^−6^	6.0	5%
**MBD1-3+ sl-Atox1 + Cu(I)**	[25 25 88]	1 × 10^−10^	5.0	30%
	[25 25 92]	1 × 10^−6^	6.0	70%
**MBD3-4**	[25 25 90]	1 × 10^−9^	5.0	98%
	[25 25 86]	1 × 10^−9^	0.0	2%
**MBD3-4 + Cu(I)**	[25 25 90]	1 × 10^−9^	5.0	85%
	[25 25 88]	0.6 × 10^−10^	0.0	15%
**MBD3-4 + sl-Atox1 + Cu(I)**	[25 25 90]	1 × 10^−9^	5.0	85%
	[25 25 88]	0.6 × 10^−10^	0.0	15%
**MBD4-6**	[25 25 88]	1 × 10^−10^	3.0	16%
	[25 25 92]	1 × 10^−6^	6.0	84%
**MBD4-6 + Cu(I)**	[25 25 88]	1 × 10^−10^	3.0	16%
	[25 25 92]	1 × 10^−6^	6.0	84%
**MBD4-6 + Atox1**	[25 25 90]	6 × 10^−9^	0.0	100%
**MBD4-6 + Atox1 + Cu(I)**	[25 25 90]	6 × 10^−9^	0.0	100%
**MBD4-6 + sl-Atox1 + Cu(I)**	[25 25 88]	1 × 10^−10^	5.0	50%
	[25 25 92]	1 × 10^−6^	6.0	50%

## Abbreviations

Cys	Cysteine
WT	Wild type
CD	Circular dichroism
EPR	Electron paramagnetic resonance
CW-EPR	Continuous wave electron paramagnetic resonance
DEER	Double electron–electron resonance
RT	Room temperature
O.N.	Overnight
sl	Spin-labeled
MBD	Metal binding domain
NMR	Nuclear magnetic resonance
SDSL	Site-directed spin labeling
MTSL	Methanesulfonothiolate
